# Spontaneous arterial rupture complicating neurofibromatosis type 1: a case report of massive lumbogluteal hematoma and literature review

**DOI:** 10.3389/fmed.2026.1792286

**Published:** 2026-05-13

**Authors:** Jing Zhang, Xin Pang, Yanan Meng, Qinlong Sun

**Affiliations:** 1 Department of General Practice, The Affiliated Hospital of Hebei University, Baoding, Hebei, China; 2 Department of Neurology, The Affiliated Hospital of Hebei University, Baoding, Hebei, China; 3 Department of Critical Care Medicine, The Affiliated Hospital of Hebei University, Baoding, Hebei, China

**Keywords:** neurofibromatosis type 1, spontaneous hemorrhage, subcutaneous hematoma, transcatheter arterial embolization, vascular complication

## Abstract

Neurofibromatosis type 1 (NF1) is a multisystem genetic disorder in which vascular dysplasia can lead to rare, life-threatening spontaneous hemorrhage. We present the case of a 62-year-old male NF1 patient who presented with acute left lumbar pain and swelling without a history of trauma or anticoagulant use. Computed tomography (CT) revealed a large hematoma in the left lumbogluteal region and a presacral mass. Active bleeding from the left second lumbar artery and the subcostal artery was confirmed by digital subtraction angiography and successfully managed with transcatheter arterial embolization (TAE). The postoperative course was complicated by hematoma rupture and secondary infection. This was controlled with ultrasound-guided drainage (yielding approximately 3000 mL of old blood), debridement, and targeted antibiotic therapy (clindamycin). This case illustrates the rare yet complete clinical trajectory of spontaneous arterial rupture in NF1, from massive hematoma formation to infectious complications. It underscores that acute pain or an unexplained mass in an NF1 patient should raise immediate suspicion for spontaneous hemorrhage, necessitating prompt vascular imaging and multidisciplinary intervention to improve outcomes.

## 1 Introduction

Neurofibromatosis type 1 (NF1) is a common autosomal dominant neurocutaneous syndrome with highly variable clinical manifestations (1). Diagnosis primarily relies on characteristic findings such as café-au-lait macules, axillary and inguinal freckling, Lisch nodules, and multiple neurofibromas (2–4). Beyond these classic features, patients with NF1 are predisposed to a spectrum of systemic complications, including learning disabilities, skeletal abnormalities, and an increased risk of benign and malignant tumors (5, 6). Vascular dysplasia represents a relatively uncommon but potentially devastating complication of NF1. NF1-associated vasculopathy can affect arteries of any size, leading to stenosis, aneurysm formation, or spontaneous rupture (7–9). Although spontaneous rupture of these dysplastic vessels is rare, it constitutes a life-threatening emergency, typically presenting with acute pain and hemorrhage into a body cavity or soft tissue (10, 11). The lumbar and subcostal arteries are among the uncommon sites for such an event. The low incidence and frequent absence of a clear traumatic history make clinical diagnosis challenging. Management requires a multidisciplinary approach, with transcatheter arterial embolization serving as the critical first-line intervention to control active bleeding. Subsequent complications, such as hematoma infection, can further complicate the clinical course, necessitating tailored management strategies. Herein, we report the case of a 62-years-old man with long-standing NF1 who presented with spontaneous rupture of the left second lumbar and subcostal arteries, resulting in a massive subcutaneous hematoma complicated by secondary infection. This case report aims to: (1) heighten awareness of spontaneous vascular rupture in the differential diagnosis of acute pain in NF1 patients; (2) underscore the pivotal role of angiography in emergency intervention; and (3) outline an integrated management approach for this severe complication.

## 2 Case report

A 62-year-old male presented with a 1-day history of left lumbar pain and swelling. He denied any recent or remote trauma to the area and was not taking anticoagulant or antiplatelet medications. He had a 30-years history of Neurofibromatosis type 1 (NF1). No other significant medical, surgical, psychiatric, or medication history was reported. His parents and children were healthy, with no family history of consanguinity or other heritable disorders.

**FIGURE 1:**
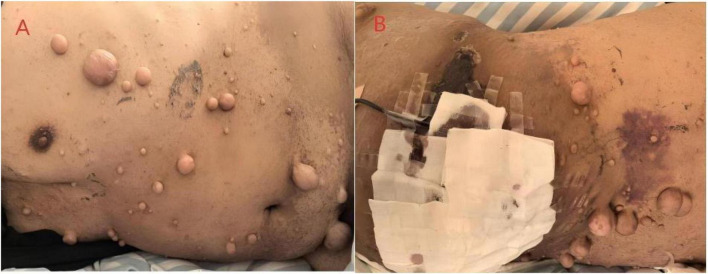
**(A,B)** Physical examination revealed multiple café-au-lait macules with well-defined borders, measuring 0.5–5.0 cm in diameter, some confluent. Multiple scattered neurofibromas (1–5 cm in diameter) were observed on the trunk and limbs. Drainage was performed for the massive lumbar hematoma.

Physical examination: Body temperature was 36.3 °C, pulse rate 94 bpm, respiratory rate 20 bpm, blood pressure 123/85 mmHg, and oxygen saturation 96% multiple café-au-lait macules with well-defined borders, measuring 0.5–5.0 cm in diameter, some confluent. Multiple scattered neurofibromas (1–5 cm in diameter) were observed on the trunk and limbs. Genitalia were normally developed. A large hematoma, approximately 18 cm × 10 cm, was evident in the left lumbogluteal region. Neurological examination was unremarkable (Figures 1A, B).

Laboratory tests on admission showed elevated white blood cell count, reduced hemoglobin and hematocrit, prolonged INR, elevated C-reactive protein and D-dimer, and normal procalcitonin (Table 1).

**TABLE 1:** Laboratory findings on admission.

Inspection item	Result	Normal range
White blood cell count (×10^9^/L)	21.06	3.5–9.5
Neutrophil count	17.5	1.8–6.3
Neutrophil (%)	83.1	40–75
Hemoglobin (g/L)	96	130–175
Erythrocrit (%)	29.5	40–50
Platelet count (×10^9^/L)	299	125–350
C-reactive protein (mg/L)	82.6	0–6
Procalcitonin (ng/ml)	0.05	0.00–0.25
Activated partial thromboplastin time (s)	31.8	28–42
Prothrombin time international normalized ratio (INR)	1.23	0.8–1.2
D-Dimer (ng/ml)	760.8	0–243

**FIGURE 2:**
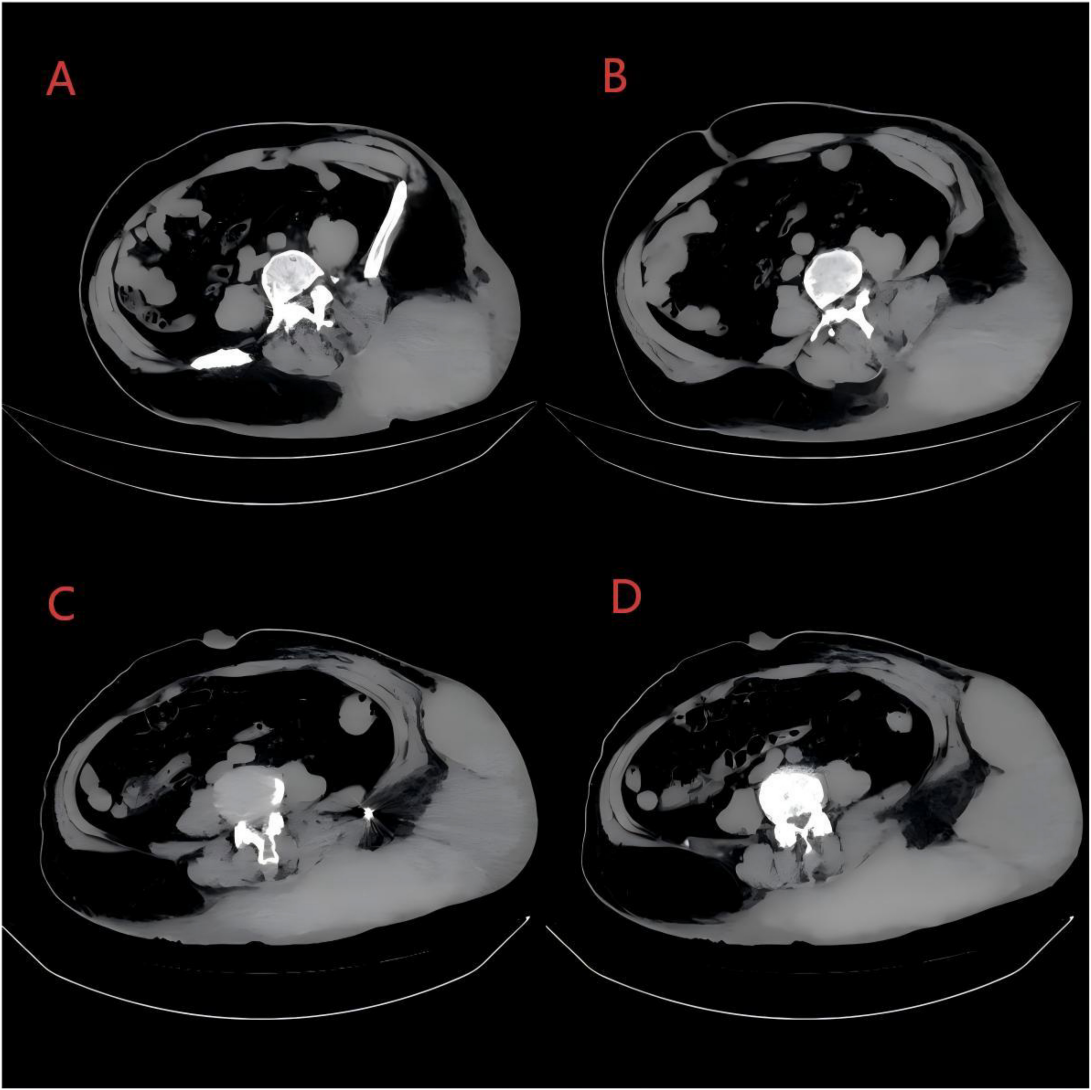
**(A,B)** Abdominal CT: there is marked skin thickening over the lower back and left gluteal region. A slightly hyperdense subcutaneous mass is noted on the left side of the lumbar region, suggestive of a possible hematoma. Contrast-enhanced imaging is recommended. A pre-sacral occupying lesion is identified, with extensive, multiple cutaneous nodules observed within the scanned field. **(C,D)** Post-embolization follow-up abdominal CT: compared to the prior lower abdominal imaging, the extent of the lesion appears to have increased, with a dense subcutaneous shadow observed on the left side of the lumbar region.

On the day of admission, computed tomography (CT) showed marked thickening of the skin over the left lumbodorsal and left gluteal regions. An irregular high-density mass measuring approximately 14.6 × 9.8 × 9.6 cm was observed in the subcutaneous tissue. Meanwhile, a soft-tissue density mass measuring approximately 4.5 × 2.7 × 3.9 cm was identified in the presacral region, with a clear boundary, homogeneous density, and a CT value of approximately 26 HU (Figures 2A,B). Its CT value was significantly lower than the density of subcutaneous hematoma (39–52 HU), and thus the presacral space-occupying lesion was considered to be a possible neurofibroma. Numerous superficial cutaneous nodules were widely distributed over the body surface. Ultrasonography revealed a cystic-solid mass in the subcutaneous tissue of the lumbogluteal region with ill-defined borders and an irregular shape.

After routine preoperative evaluation on the same day, emergency angiography was performed by interventional radiologists. Contrast extravasation was noted from both the left second lumbar artery and the left subcostal artery, consistent with active bleeding. The patient presented with active bleeding, and received transfusion of 2 units of red blood cells and approximately 1500 mL of crystalloid fluid. Following blood transfusion and fluid resuscitation, hemodynamic stability was achieved without the need for vasoactive medications. Transcatheter arterial embolization (TAE) was successfully performed for both bleeding vessels. CT reexamination at 3 days showed enlargement of the subcutaneous hematoma (Figures 2C,D).

On postoperative day 3, rupture and exudation occurred at the hematoma site. Secretion samples were collected for bacterial culture and drug susceptibility testing, and anti-infective therapy with piperacillin-tazobactam was initiated. On the 6th postoperative day, the secretion culture yielded hemolytic *Staphylococcus*, and the drug sensitivity test showed susceptibility to clindamycin. Accordingly, the antibiotic regimen was adjusted to clindamycin for anti-infective treatment.

In this patient with neurofibromatosis type 1 (NF1), the hematoma was extensive. Given that patients with NF1 are characterized by vascular dysplasia and a high likelihood of widespread rupture and hemorrhage from microvessels, early and extensive hematoma evacuation may lead to recurrent hemorrhage following decompression. To ensure further hemostatic stability after embolization, delayed drainage was performed. On postoperative day 16 (day 16 after admission), re-examination by ultrasound revealed a huge cystic-solid mass in the left lumbar and gluteal subcutaneous tissue, with a maximum depth of approximately 18.6 cm and multiple hyperechoic septations inside. Color Doppler flow imaging (CDFI) demonstrated blood flow signals within the septations. Ultrasound-guided catheter drainage was performed, yielding approximately 3000 mL of dark red old hematoma.

On postoperative day 20, inadequate drainage was noted due to suspected blood clots, conferring a high risk of secondary infection. Therefore, debridement surgery was performed. Approximately 200 mL of organized clot was noted within the wound cavity.

At the 3-months follow-up, the patient’s lumbodorsal pain and swelling had completely resolved. No mass was palpable in the left lumbar and gluteal region, and the patient had full, unimpaired limb mobility. Repeat ultrasound of the left lumbar and gluteal subcutaneous soft tissues showed no evidence of mass or fluid collection. Laboratory examinations revealed no signs of inflammation or abnormal coagulation function (White blood cell count 8.9 × 109/L, Neutrophil (69%), Hemoglobin 119 g/L, C-reactive protein 5 mg/L, Procalcitonin 0.09 ng/ml). The necessity of lifelong surveillance, including a specific schedule, was thoroughly discussed with the patient. He expressed high satisfaction with the treatment outcome and agreed to adhere to the recommended follow-up plan.

## 3 Discussion

This case report describes a rare emergency in a 62-years-old NF1 patient: a massive hematoma complicated by secondary infection, resulting from spontaneous rupture of the lumbar and subcostal arteries. It comprehensively illustrates the management of this condition, from initial recognition of acute hemorrhage to angiographic embolization, hematoma drainage, and infection control. This experience highlights both the diagnostic and therapeutic challenges posed by this severe, yet frequently overlooked, vascular complication of NF1, and underscores the critical importance of a multidisciplinary approach.

### 3.1 Pathophysiology and clinical challenges of NF1 vasculopathy

Vascular pathology in NF1 is multifactorial, involving processes that lead to a fragile arterial wall prone to aneurysm, stenosis, or rupture (12). Commonly affected sites are renal, mesenteric, and cerebral arteries. Pathogenesis relates to deficient neurofibromin function (13). Large vessels may rupture from neurofibroma compression; small vessels show dysplastic fibrohyaline thickening, causing stenosis and focal weakness (14, 15). Spontaneous rupture, though rare (0.4%–6.4%), is often fatal (16). A total of 10 cases of lumbar artery and/or intercostal artery rupture in neurofibromatosis type 1 (NF1) were retrieved via literature search (17–26) (Table 2).

**TABLE 2:** Summary of previous cases and the present case of neurofibromatosis type 1 with rupture of intercostal artery and/or lumbar artery.

Ref	Author	Year	Age	Sex	Symptoms	Hemodynamic status	Bleeding site
(17)	Kipfer B	2001	42	Female	Chest pain	Stable	Right hemothorax
(18)	Miura T	2005	46	Male	Back pain	Shock	Left hemothorax
(19)	Matsumoto I	2007	49	Male	Back pain	Shock	Left hemothorax
(20)	Aizawa K	2010	48	Male	Back pain	Stable	Right hemothorax
(21)	Misao T	2012	40	Male	Back pain	Stable	Right spontaneous hemothorax
(22)	Keerati Hongsakul	2013	46	Female	Chest pain	Stable	Left hemothorax
(23)	Ishigaki T	2017	52	Male	Back pain	Stable	Retroperitoneal hematoma
(24)	Overgaard EK	2020	49	Male	Back pain	Stable	Retroperitoneal hematoma
(25)	Ken Tsuchida	2023	55	Female	Back pain	Stable	Retroperitoneal hematoma
(26)	Kanda T	2026	65	Female	Back pain	Shock	Right hemothorax, posterior mediastinal hematoma
Present case		2025	62	Male	Back pain	Stable	Subcutaneous hematoma

Herein, we report a 62-year-old male patient with a 30-years history of NF1, who had no prior use of anticoagulant or antiplatelet agents, nor a history of trauma or surgery. He presented to our hospital with the chief complaint of left lumbar pain accompanied by swelling. Digital subtraction angiography (DSA) demonstrated rupture and hemorrhage of the left second lumbar artery and subcostal artery; no aneurysm or vascular compression by neurofibromas was identified. Spontaneous rupture was considered to be caused by intrinsic pathological changes of the arterial wall. This condition is characterized by an insidious onset and massive bleeding, which renders early diagnosis and treatment challenging. As paired segmental vessels supplying vital retroperitoneal areas, their rupture risks major hemorrhage. The concurrent involvement of two adjacent arteries highlights the diffuse, systemic nature of NF1 vasculopathy beyond isolated anomalies (22).

The clinical difficulty lay in interpreting a common symptom–acute localized pain with swelling–in the context of a rare, spontaneous event. This presentation is non-specific, overlapping with conditions like renal colic or muscle injury. In a patient with overt NF1 features, diagnostic consideration may be prematurely anchored to neurofibroma-related complications. The unusual location of the bleed (lumbar arteries), compared to more common sites for NF1 vasculopathy, further challenges timely diagnosis. This case thus emphasizes that managing NF1 requires vigilance for its unpredictable vascular manifestations, even when symptoms seem typical of more benign processes.

### 3.2 Multidisciplinary staged management: a strategy integrating interventional, surgical, and medical care

Stage one: interventional embolization for acute hemorrhage control

The definitive identification of actively bleeding vessels requires dynamic contrast-enhanced CT angiography (CTA) or digital subtraction angiography (DSA) (27–29). Given the characteristic fragility of vessels in NF1 patients, surgical hemostasis is often challenging; therefore, endovascular intervention should be the primary consideration when arterial rupture is suspected. This patient presented with neurofibromatosis type 1 (NF1) complicated by arterial hemorrhage, accompanied by arterial wall dysplasia, which increases the risk of iatrogenic injury during interventional embolization. Microcatheterization and gentle interventional techniques are the preferred therapeutic approaches (30–33). It allows for the rapid and precise occlusion of culprit vessels, stabilizes hemodynamics, minimizes tissue trauma, and secures a critical window for subsequent management (34).

Stage two: strategic management of the hematoma

While embolization secures hemostasis, the resultant massive hematoma itself poses a subsequent management challenge. Its substantial size–exemplified by the 18.6 cm deep collection in our report–carries risks of local compression and infection, mandating active management. Image-guided drainage (yielding ∼3000 mL here) is the primary non-surgical approach. Surgical indications include TAE failure, hematoma-induced organ compromise (e.g., hemothorax), or infection. Organized, septated hematomas may require late surgical evacuation for definitive healing.

Stage three: wound management and infection control

When referring to skin and soft tissue infections (SSTIs), the most relevant pathogens are *Staphylococcus aureus* and *Streptococcus pyogenes* (35). Gram-negative bacilli represent another important group of bacteria, particularly in hospitalized patients. For empirical antibiotic selection in patients with necrotizing soft tissue infection, broad-spectrum β-lactam antibiotics (such as piperacillin-tazobactam) are the first-line choice (36). Large retroperitoneal or subcutaneous hematomas are prone to secondary infection, as accumulated blood provides an ideal medium for bacterial growth. To date, the vast majority of studies on neurofibromatosis complicated by arterial rupture (Table 3) have employed transcatheter arterial embolization (TAE) as the therapeutic strategy. For patients with concurrent infection, debridement and drainage and/or combined antibiotic therapy were employed. In our case, *Staphylococcus hemolyticus* was cultured, which is regarded as an opportunistic pathogen. The administration of clindamycin guided by antibiotic susceptibility testing embodies the principle of precision anti-infective therapy. Notably, the integrated treatment paradigm of “minimally invasive drainage + precision anti-infection + stepwise debridement” effectively achieved local infection control and precluded the progression to sepsis. It is noteworthy that NF1 patients carry specific risks for delayed wound healing and infection due to cutaneous neurofibromas and underlying microangiopathy (37). This necessitates meticulous wound care and, when indicated, consideration of advanced reconstructive options such as negative pressure wound therapy or flap repair. However, an in-depth evaluation of immune function and microcirculatory status was not performed in this NF1 patient. Although the risk of delayed wound healing was noted, the specific mechanisms underlying increased susceptibility to infection were not elucidated by relevant examinations, such as immune cell subset analysis and microcirculatory blood flow monitoring. Consequently, it is difficult to develop targeted and individualized therapeutic interventions.

**TABLE 3:** A study on the treatment and prognosis of NF1 complicated with rupture of lumbar/subcostal artery and secondary infection of hematoma.

Ref	Hemorrhagic artery	Treatments	Complication	Outcome
(17)	An intercostal artery	TAE + Thoracoscopic evacuation	None	Alive
(18)	The right 7th intercostal artery	TAE + Chest tube irrigation + Intravenous antibiotic therapy	Empyema	Alive
(19)	The left 11th intercostal artery	TAE + Closed thoracic drainage	ND	Alive
(20)	The right 11th intercostal artery	Thoracotomy for hemostasis	ND	Alive
(21)	The right 10th intercostal artery the left subcostal artery	TAE	None	Alive
(22)	The left 5th intercostal artery	TAE + Closed thoracic drainage	None	Alive
(23)	The right lumbar artery	TAE	None	Alive
(24)	The right lumbar artery	TAE	None	Alive
(25)	The right lumbar artery	TAE	None	Alive
(26)	The right 9th intercostal artery	TAE	None	Alive
Present case	The left second lumbar artery the left subcostal artery	TAE + Minimally invasive drainage + Intravenous antibiotic therapy	Soft tissue infection	Alive

Ref., reference; ND, not described; TAE, transcatheter arterial embolization.

### 3.3 Long-term follow-up: a clinical imperative

Neurofibromatosis type 1 is, by nature, a multifocal and progressive disease (4, 38). This disease carries a substantial risk of systemic vascular dysplasia, and is commonly associated with occult intracranial and cervical vascular lesions, including asymptomatic stenosis and aneurysms. Therefore, lifelong patient education and surveillance are paramount. This adult high-risk NF1 patient with a history of TAE warranted intensive lifelong vascular monitoring: daily blood pressure measurement, complete blood count, targeted ultrasound. One-time computed tomographic angiography (CTA) of the involved vessels and entire aorta–including the iliac, renal, mesenteric, cerebral, and carotid arteries-should be obtained at 6 and 12 months after discharge to exclude occult pseudoaneurysms. Baseline head and neck magnetic resonance angiography (MRA) is also recommended during long-term follow-up for early detection of asymptomatic vascular stenosis or aneurysms. A multidisciplinary follow-up program should be implemented to enable early detection of rebleeding, *de novo* aneurysms, and other adverse vascular events. If no disease progression or related symptoms develop by 1 year post-discharge, surveillance may be reduced to annual intervals and continued lifelong to optimize long-term prognosis. In our case, the patient understood and accepted this lifelong follow-up plan, establishing a critical foundation for long-term prognosis.

### 3.4 Clinical implications and future perspectives

Patients with neurofibromatosis type 1 (NF1) present with cutaneous neurofibromas involving multiple body sites and associated microvascular abnormalities. Open surgery is associated with an increased risk of wound bleeding, delayed healing, and infection. The stepwise strategy adopted in this case-interventional hemostasis, minimally invasive drainage, and debridement when necessary-is more appropriate for this condition.

Currently, no unified criteria exist for evaluating the risk of vascular events in patients with NF1 clinically. Moving forward, multi-center, large-cohort studies are needed, which should include relevant factors such as patient age, NF1 disease duration, prior vascular event history, comorbid conditions (e.g., hypertension, diabetes mellitus), imaging findings (unexplained space-occupying lesions, effusions), and laboratory parameters (D-dimer, fibrinogen). These studies will help identify independent risk factors for vascular complications, develop a quantitative risk scoring model, enhance the relevance and efficiency of clinical management, and facilitate the shift from empirical to precision-based diagnosis and treatment of NF1-related vascular complications.

## 4 Conclusion

This case provides a rare, complete illustration of spontaneous lumbar and subcostal artery rupture leading to a massive hematoma and secondary infection in a patient with neurofibromatosis type 1 (NF1). It highlights a key clinical pearl: spontaneous vascular rupture must be ruled out first in NF1 patients with acute pain/mass. Diagnosis hinges on prompt CTA/DSA. Treatment follows a logical sequence: embolize, drain the hematoma (surgically if needed), then manage infection and wound healing with NF1-specific considerations. Lifelong monitoring is advised due to recurrence risk. Our experience adds to the understanding of NF1 vasculopathy, demonstrates a multidisciplinary management model, and calls for broader awareness of NF1’s systemic risks. Future studies should focus on risk stratification and optimizing long-term care.

## Data Availability

The raw data supporting the conclusions of this article will be made available by the authors, without undue reservation.
